# Prophylactic defunctioning stomas improve clinical outcomes of anastomotic leak following rectal cancer resections: An analysis of the US Rectal Cancer Consortium

**DOI:** 10.1007/s00384-024-04600-3

**Published:** 2024-03-18

**Authors:** Katherine Hrebinko, Vincent P. Anto, Katherine M. Reitz, Adriana C. Gamboa, Scott E. Regenbogen, Alexander T. Hawkins, M. Benjamin Hopkins, Aslam Ejaz, Philip S. Bauer, Paul E. Wise, Glen C. Balch, Jennifer Holder-Murray

**Affiliations:** 1 Department of Surgery, University of Pittsburgh Medical Center, Pittsburgh, USA; 2 Division of Vascular Surgery, Department of Surgery, University of Pittsburgh Medical Center, Pittsburgh, USA; 3 Division of Surgical Oncology, MD Anderson Cancer Center, University of Texas, Austin, USA; 4 Division of Colorectal Surgery, Department of Surgery, University of Michigan, Ann Arbor, USA; 5 Section of Colon & Rectal Surgery, Division of General Surgery, Vanderbilt University Medical Center, Nashville, USA; 6 Division of Surgical Oncology, Department of Surgery, The Ohio State University, Columbus, USA; 7 Department of Surgery, Allegheny Health Network, Pittsburgh, USA; 8 Section of Colon & Rectal Surgery, Department of Surgery, Washington University in St. Louis School of Medicine, St. Louis, USA; 9 Division of Colon & Rectal Surgery, Department of Surgery, Emory University, Atlanta, USA; 10 Division of Colon and Rectal Surgery, Department of Surgery, University of Pittsburgh Medical Center, Kaufmann Medical Office Building, Suite 603, 3471 Fifth Avenue, Pittsburgh, PA 15213 USA

**Keywords:** Rectal cancer, Anastomotic leak, Fecal diversion, Clinical outcomes

## Abstract

**Purpose:**

Anastomotic leak (AL) is a complication of low anterior resection (LAR) that results in substantial morbidity. There is immense interest in evaluating immediate postoperative and long-term oncologic outcomes in patients who undergo diverting loop ileostomies (DLI). The purpose of this study is to understand the relationship between fecal diversion, AL, and oncologic outcomes.

**Methods:**

This is a retrospective multicenter cohort study using patient data obtained from the US Rectal Cancer Consortium database compiled from six academic institutions. The study population included patients with rectal adenocarcinoma undergoing LAR. The primary outcome was the incidence of AL among patients who did or did not receive DLI during LAR. Secondary outcomes included risk factors for AL, receipt of adjuvant therapy, 3-year overall survival, and 3-year recurrence.

**Results:**

Of 815 patients, 38 (4.7%) suffered AL after LAR. Patients with AL were more likely to be male, have unintentional preoperative weight loss, and are less likely to undergo DLI. On multivariable analysis, DLI remained protective against AL (*p* < 0.001). Diverted patients were less likely to undergo future surgical procedures including additional ostomy creation, completion proctectomy, or pelvic washout for AL. Subgroup analysis of 456 patients with locally advanced disease showed that DLI was correlated with increased receipt of adjuvant therapy for patients with and without AL on univariate analysis (SHR:1.59; [95% CI 1.19–2.14]; *p* = 0.002), but significance was not met in multivariate models.

**Conclusion:**

Lack of DLI and preoperative weight loss was associated with anastomotic leak. Fecal diversion may improve the timely initiation of adjuvant oncologic therapy. The long-term outcomes following routine diverting stomas warrant further study.

## Introduction

Despite increasing evidence to support neoadjuvant therapy followed by watch-and-wait protocols in certain rectal cancer patients, many current standard-of-care pathways continue to include surgical resection [1]. Low anterior resection is one sphincter-sparing operative approach to rectal cancer that involves resection of the rectum along with its lymphovascular pedicle and creation of an anastomosis. Anastomotic leak (AL) is a feared complication of low anterior resection (LAR) resulting in significant morbidity including reoperation, prolonged hospitalization, pelvic sepsis, intensive care unit admission, long-term bowel dysfunction, and permanent stoma creation [2, 3]. It is therefore imperative that risk factors for AL be identified and protective measures, such as fecal diversion, be instituted to mitigate the risk of this complication. Although the consequences of anastomotic leak on perioperative outcomes are well-established, the impact of anastomotic leak on long-term oncologic outcomes is conflicting in the literature [4–6].

The association of protective stoma on the incidence of AL has been evaluated by several large, randomized control trials and meta-analyses. Defunctioning stomas appear to significantly reduce the rates of symptomatic anastomotic leaks and reoperations [7–10]. Oncologic outcomes related to defunctioning stomas have not been extensively studied. Diverting stomas are not without their own inherent complications including dehydration, electrolyte abnormalities, and surgical complications [11–14]. Stoma reversal requires a return to the operating room with hospital admission and may never be completed, particularly in frail or elderly patients [15].

Using the granular, real-world data available in the United States Rectal Cancer Consortium (USRCC), we aimed to understand factors associated with AL and evaluate the relationship of AL with oncologic outcomes.

## Materials and methods

### Checklist for the reporting of observational studies

This article was written in adherence with the Strengthening the Reporting of Observational Studies in Epidemiology (STROBE) checklist for the reporting of observational studies [16].

### Data source and study design

After obtaining Institutional Review Board approval, patients were identified from the USRCC, a national repository of rectal cancer outcomes compiled from six academic medical institutions including the University of Pittsburgh Medical Center, Emory University, Vanderbilt University Medical Center, University of Michigan, Washington University School of Medicine, and The Ohio State University. The USRCC includes all patients diagnosed with rectal cancer from 2007 to 2017 cared for at the included institutions. Information regarding recurrence and survival was collected until the date of last follow-up for all patients. Using these data, we evaluated the correlation and association between AL, pre- and intraoperative characteristics, survival, and oncologic outcomes. We then evaluated the association between pertinent risk factors on the receipt of postoperative adjuvant therapy among patients meeting evidence-based guidelines for such therapies. All analyses were completed using Stata Statistical Software (StataCorp. 2019. *Stata Statistical Software*: Release 16. College Station, TX: StataCorp LLC).

### Patient selection

**Fig. 1:**
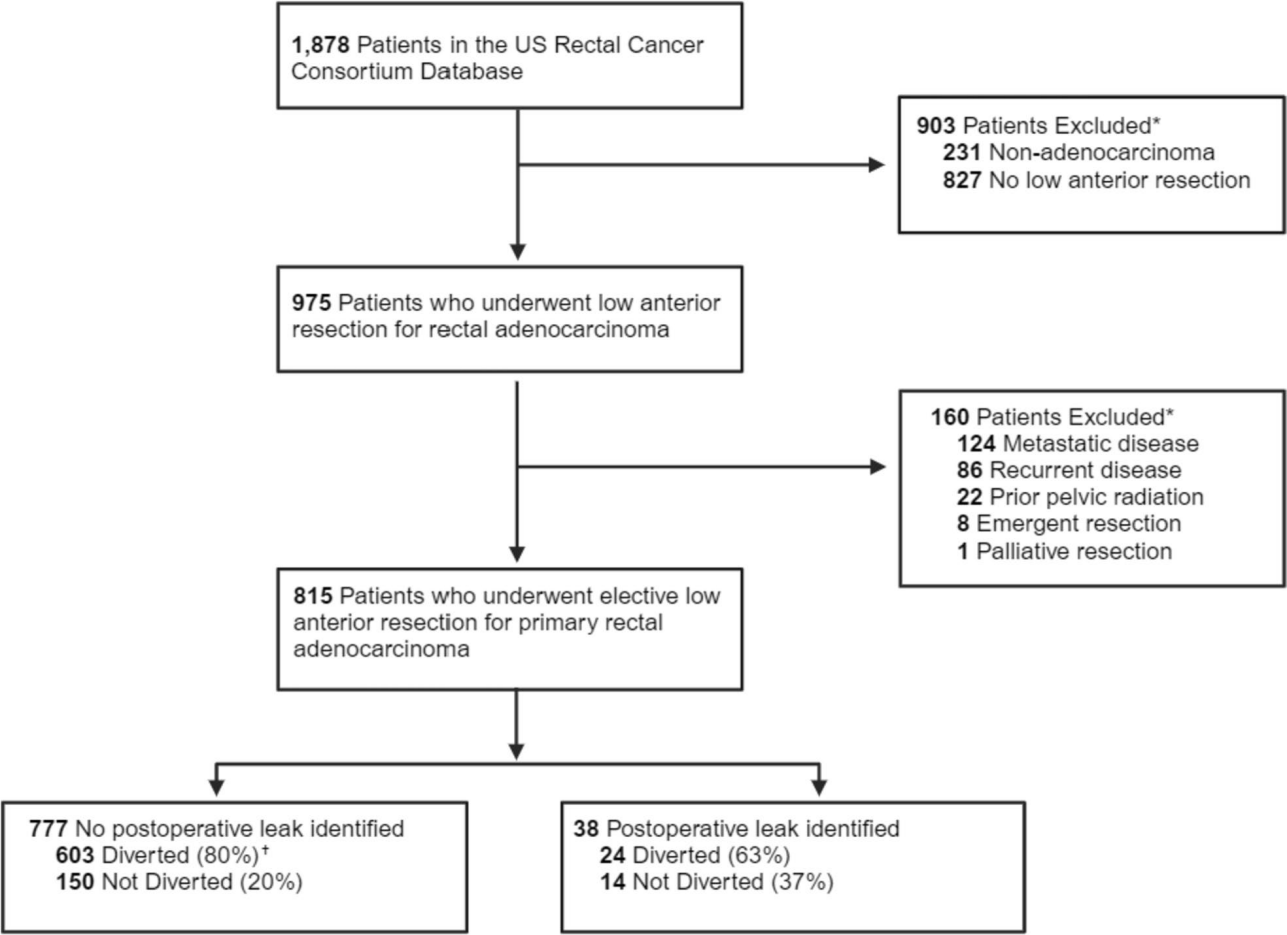
Flowchart of patient selection. ^*^Indicating number of patients excluded, subgroups demonstrate total numbers as conditions are not mutually exclusive. ^✝^Total not 777 due to patients (*n* = 24) lacking diversion status at index operation

All patients who underwent LAR with curative intent for pathologically confirmed primary rectal adenocarcinoma were identified (Fig. 1). Patients with pathology other than adenocarcinoma on pathologic analysis or those relegated to watch-and-wait, local transanal excision, or abdominoperineal resection were excluded. Additionally, patients undergoing emergent surgery, those with recurrent rectal cancer, history of prior pelvic irradiation before rectal cancer treatment, and metastatic disease at diagnosis were excluded from the analysis. Patient demographic, clinicopathologic, and treatment characteristics, as well as information regarding postoperative outcomes including complications, survival, recurrence, and receipt of adjuvant therapy, were obtained from the review of each institutional electronic medical record.

### Definitions

Clinical and pathologic staging was based on the American Joint Committee on Cancer (AJCC) 8th edition staging guidelines [17]. Comorbidity burden was assessed by the American Society of Anesthesiologists’ (ASA) classification of physical health. Preoperative weight loss has been implicated in complication rates in colorectal surgery. This study utilized a cutoff for unintentional weight loss of more than 10% of the patient’s usual body weight over the 6 months prior to surgery, similar to previous works [18, 19]. Postoperative complications were classified based on the 7-category Clavien-Dindo scale [20]. Locally advanced disease was defined as patients with T stage 3–4 or N stage 1–2 disease on either preoperative staging workup or final pathologic analysis following resection. Tumor location refers to the pre-treatment relationship of the rectal tumor to the anal sphincter complex.

### Outcomes

The primary outcome of interest was the incidence of AL, which was defined as leakage of luminal contents from the surgical staple or suture line diagnosed clinically or radiographically within 12 months of rectal tumor resection, among patients with or without diverting loop ileostomy (DLI) at the time of LAR. Granular information for patients suffering AL was collected, including method of leak diagnosis, time to diagnosis, time to intervention, and method of intervention. Secondary outcomes included 1-year receipt of adjuvant chemotherapy, 3-year overall survival (OS), and 3-year rectal cancer recurrence (locoregional or metastatic) with mortality as a competing risk. The 3-year time horizon was determined by the average time to last follow-up to mitigate effects of loss to follow-up.

### Statistical analysis

Categorical variables were presented as number (%) and continuous variables as mean (standard deviation (SD)) or median (interquartile range (IQR)). Patients were stratified into cohorts based on the presence or absence of postoperative AL. Group characteristics were compared between cohorts using Chi-Square or Fisher’s exact tests for categorical variables and *t*-tests, Kruskal–Wallis, or Wilcoxon rank-sum tests for continuous variables, when appropriate.

Survival analysis was utilized to evaluate all outcomes. Mortality was compared between groups with Kaplan–Meier curves and associated log-rank testing. The risk of various outcomes was assessed with Fine-Gray models, controlling for the competing risk of mortality, censoring at last known follow-up, and clustering at the hospital level. These data were presented with cumulative hazard curves and expressed as sub-distribution hazard ratios. Multivariate analyses were carried out with two serial models. Analyses were first limited to demographic and clinical variables that were significantly different between groups on univariate analysis to avoid overfitting the model given the small number of AL events. Analyses were then expanded to other clinically relevant variables [21]. Both models were presented to demonstrate consistency across point estimates and the associated variability with an increasing number of covariates per AL event. All variables were assessed for missingness. Variables with > 10% missingness were excluded from any multivariate analyses. Patients suffering AL were further stratified by the presence or absence of DLI during their index operation to determine the relationship of fecal diversion on perioperative and oncologic outcomes. Lastly, subgroup analysis was performed among patients meeting the criteria for administration of adjuvant therapy under NCCN guidelines, including patients with clinical and/or pathologic stage II and III disease [22]. Proportional hazard assumptions were evaluated, as appropriate. A two-tailed *p*-value < 0.05 was designated as the cutoff for statistical significance.

## Results

### Cohort characteristics

The USRCC includes 1,878 patients with rectal cancer, of which 815 met the inclusion criteria (Fig. 1). Patient demographics included a mean age of 58.4 (SD: $\pm$ 12.3) years, 60.4% male, and 89.9% white (Table 1). The median duration of follow-up was 3.02 (IQR: 1.86–4.67) years. Among this group, 38 (4.7%) patients suffered a postoperative AL. Patients with AL were more likely to be male (AL: 78.9% vs. no AL: 59.5%, *p* = 0.02), had less prior abdominal surgery (AL: 6.1%, no AL: 30.1%, *p* = 0.02), had more preoperative unintentional weight loss (AL: 24.3%, no AL: 13.3%, *p* = 0.049), and received less fecal diversion procedures at their index operation (AL: 63.2%, no AL: 80.1%, *p* = 0.01) (Table 1). Patients with AL had more reoperations (AL: 47.4%, no AL: 5.7%, *p* < 0.001) and more readmissions (AL: 78.9%, no AL: 25.5%, *p* < 0.001) when compared to no AL.

**Table 1:** Comparison of patient, tumor, and treatment characteristics between cohorts

	No AL *N* = 777	AL *N* = 38
Age, years, mean (SD)	58.40 (12.4)	57.84 (9.4)
Gender: male, *n* (%)	462 (59.5%)	30 (78.9%)
Race		
White, *n* (%)	696 (89.7%)	35 (94.6%)
Black, *n* (%)	60 (7.7%)	2 (5.4%)
Other, *n* (%)	20 (2.6%)	0 (0.0%)
BMI		
< 30 kg/m^2^, *n* (%)	433 (81.0%)	23 (61.5%)
≥ 30 kg/m^2^, *n* (%)	277 (39.1%)	15 (39.5%)
Smoking within 1 month of surgery, *n* (%)	184 (24.0%)	11 (28.9%)
Insurance status		
Uninsured	28 (3.8%)	0 (0.0%)
Government-sponsored	263 (36.1%)	11 (28.9%)
Private	437 (60.0%)	27 (71.1%)
ASA classification		
1–2	354 (49.3%)	20 (52.6%)
3	357 (49.7%)	17 (44.7%)
4–5	7 (1.0%)	1 (2.6%)
Prior history of malignancy, *n* (%)	52 (6.6%)	2 (5.4%)
Inflammatory bowel disease, *n* (%)	5 (1.2%)	0 (0.0%)
Preoperative weight loss, *n* (%)	98 (13.3%)	9 (24.3%)
Prior history of abdominal operations, *n* (%)	31 (31.1%)	2 (6.1%)
Distance from anal verge, cm, median (IQR)	8.4 (6.0–10.0)	8.0 (5.0–11.0)
Tumor location, *n* (%)		
Lower rectum	204 (28.9%)	12 (33.3%)
Middle rectum	346 (49.0%)	15 (41.7%)
Upper rectum	156 (22.1%)	9 (25.0%)
Treatment characteristics		
Neoadjuvant chemoradiation, *n* (%)	568 (73.2%)	28 (73.7%)
Neoadjuvant chemoradiation completion, *n* (%)	169 (25.3%)	5 (23.8%)
Time from neoadjuvant to surgery, weeks, mean (SD)	10.74 (6.5)	9.73 (4.7%)
Operative characteristics		
Operative time, minutes, median (IQR)	220 (180–278)	242 (193–327)
Diverting loop ileostomy, *n* (%)	603 (79.2%)	24 (63.2%)
Estimated blood loss, milliliters, median (IQR)	200 (100–300)	135 (100–35)
Pathologic characteristics		
Pathologic overall stage, *n* (%)		
0	14 (13.7%)	5 (13.9%)
I	241 (31.8%)	9 (25.0%)
II	175 (23.1%)	9 (25.0%)
III	33 (4.4%)	13 (36.1%)
Histologic grade, *n* (%)		
Well differentiated	16 (3.3%)	2 (7.1%)
Moderately differentiated	433 (90.0%)	25 (89.3%)
Poorly differentiated	32 (6.7%)	1 (3.6%)
Lymphovascular invasion, *n* (%)	132 (20.6%)	9 (25.7%)
Perineural invasion, *n* (%)	83 (13.2%)	6 (18.2%)
Negative resection margins, *n* (%)	738 (96.0%)	38 (100.0%)
Complete total mesorectal excision, *n* (%)	573 (91.4%)	30 (93.8%)
Pathologic response, *n* (%)		
Complete response	74 (13.6%)	6 (23.1%)
Partial response	356 (65.4%)	14 (53.8%)
No response	114 (21.0%)	6 (23.1%)
Perioperative outcomes		
Clavien-Dindo complication score		
I	94 (33.9%)	4 (10.8%)
II	118 (42.6%)	4 (10.8%)
IIIa	27 (9.7%)	12 (32.4%)
IIIb	29 (10.5%)	15 (40.5%)
IVa	6 (2.2%)	1 (2.7%)
IVb	2 (0.7%)	1 (2.7%)
V	1 (0.4%)	0 (0.0%)
Reoperation for any complication, *n* (%)	44 (5.7%)	18 (47.4%)
Postoperative ICU admission, *n* (%)	36 (6.9%)	5 (14.7%)
ICU length of stay, days, mean (SD)	2.4 (3.15)	3.8 (2.59)
Length of stay, days, mean (SD)	6.6 (4.4)	8.3 (6.4)
30-day readmission, *n* (%)	177 (25.5%)	30 (78.9%)

*AL* anastomotic leak, *BMI* body mass index, *ASA* American Society of Anesthesiologists, *ICU* intensive care unit

### Factors associated with AL, survival, and recurrence

**Fig. 2:**
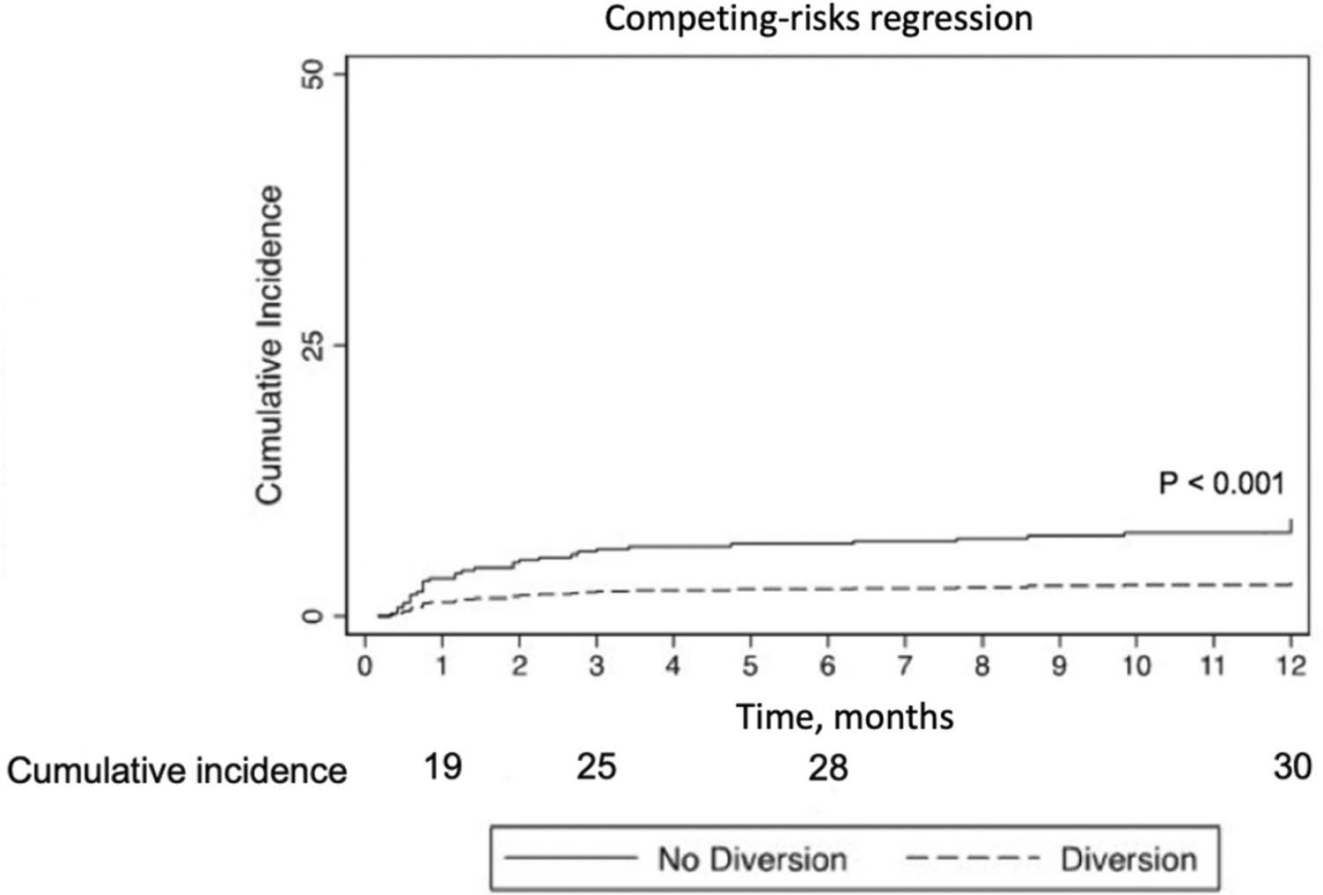
Incidence of AL over time by diversion status. Cumulative incidence of AL over time in the first year post low anterior resection stratified by patients with diverting loop ileostomy (solid line) and without diverting loop ileostomy (dashed line

**Fig. 3:**
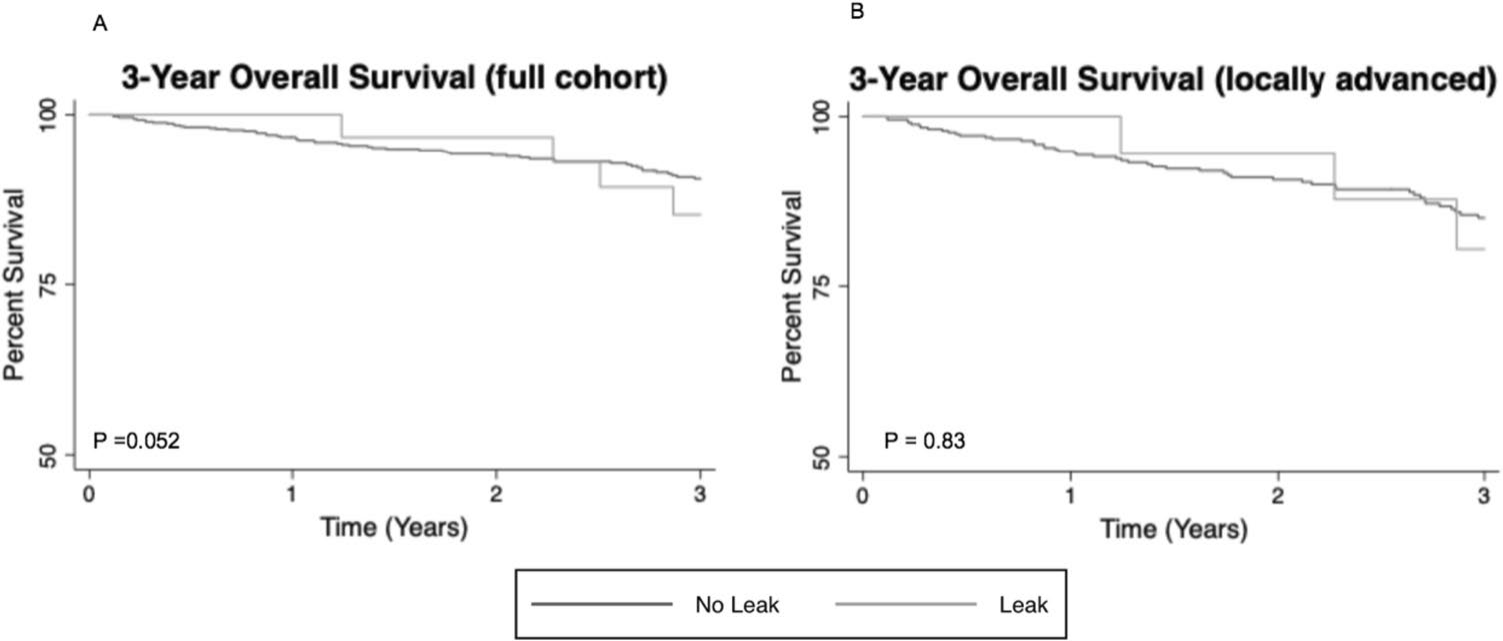
Three-year overall survival in patients with and without AL. Kaplan Meier curves illustrating overall survival (OS) over 3 years post-resection. Light gray lines represent patients without AL, and dark gray lines represent patients with AL. **A** Three-year OS for all patients included in this study stratified by presence or absence of AL. **B** Three-year OS for the patients with locally advanced (clinical stages II-III) disease stratified by presence or absence of AL

On univariate analysis, male sex (sub-hazard ratio (SHR): 2.42, 95% CI: 1.59–3.69) and preoperative weight loss (SHR: 1.82, 95% CI: 1.12–2.96) increased the risk of AL, while DLI (SHR: 0.46, 95% CI: 0.30–0.71) decreased the risk of AL when controlling for the competing risk of death. On multivariable analysis, DLI continued to decrease the risk of AL (*p* < 0.001) (Table 2, Fig. 2). There was no difference in 3-year OS (*p* = 0.052) (Fig. 3A) or 3-year recurrence at any site with death as a competing risk (SHR: 0.94, 95% CI: 0.76–1.16).

**Table 2:** Risk of anastomotic leak controlling for the competing risk of death, clustered at the institutional level

	Multivariate analysis A			Multivariate analysis B^a^		
	SHR	95% CI	*p*-value	SHR	95% CI	*p*-value
DLI (ref = no DLI)	0.42	0.27–0.65	** < 0.001**	0.36	0.23–0.57	** < 0.001**
Age	1.0–0	0.98–1.02	0.73	0.98	0.98–1.02	0.85
Male sex (ref = female sex)	2.59	1.70–3.95	** < 0.001**	1.43	1.43–3.81	**0.001**
Weight loss (ref = no preoperative weight loss)				0.88	0.88–2.95	0.12
Neoadjuvant CRT (ref = no neoadjuvant CRT)				0.67	0.67–2.44	0.45
Active smoking (ref = non-smokers)				0.46	0.46–2.78	0.80

Bolded values indicate statistical significance

*DLI* diverting loop ileostomy, *SHR* subhazard ratio, *CI* confidence interval, *CRT* chemoradiation, *ref* reference

^a^Given the limited number of AL events, Fine-Gray models were created with a limited number of variables that were significant on univariate analysis to avoid overfitting (analysis A). A second analysis (analysis B) included a greater number of clinically relevant variables

### Analysis of AL by fecal diversion status

Given the strong association between AL and DLI on multivariate analysis, the cohort of patients suffering from AL was further stratified by fecal diversion at the time of index operation. Of the patients with AL, 63.2% received a DLI at the index operation. Comparing patients with or without fecal diversion, those with DLI had an AL rate of 3.8% (24 of 627), and those without DLI had an AL rate of 8.5% (14 of 1164). There was no difference in the rate of intervention for AL among patients with or without fecal diversion (DLI: 83.0%, no DLI: 86.0%, *p* = 0.85); however, there was a significant difference in the method of intervention.

During the first year following LAR, patients with DLI at the index operation who experienced an AL were less likely to undergo subsequent operations of colostomy (DLI: 4.2%, no DLI: 21.4%, *p* = 0.01), ileostomy (DLI: 0.0%, no DLI: 45.0%, *p* = 0.01), completion proctectomy (DLI: 0.0%, no DLI: 18.0%, *p* = 0.01), or pelvic washout (DLI: 0.0%, no DLI: 9.0%, *p* = 0.01). Instead, patients with AL with DLI at the index operation were more likely to undergo percutaneous drain placement (DLI: 64.0%, no DLI: 18.0%, *p* = 0.01) or operative repair of anastomosis (DLI: 14.0%, no DLI: 0.0%, *p* = 0.01). Patients with DLI at the time of LAR who suffered AL were not more likely to undergo ostomy reversal compared to those who were diverted as intervention for AL (80.0% vs. 71.0%, *p* = 0.64).

**Fig. 4:**
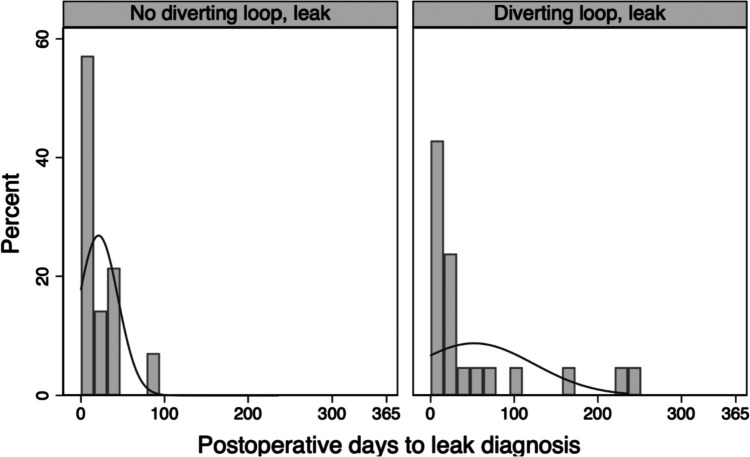
Percentage of AL diagnosed by time from surgery. Percentage of all patients with AL diagnosed at different time points from surgery. The solid line represents a normal curve overlay

Among patients with AL, median time to diagnosis and treatment did not significantly differ by sex, unintentional weight loss, or DLI; however, there was a trend towards longer times to diagnosis in days (DLI: 23.5 [9.0–76.1], no DLI: 14.5 [7.0–32.0]) and treatment in days (DLI: 29 [11.3–103.0], no DLI: 13.0 [6.5–28.0]) in patients with AL with DLI (Fig. 4). For patients with DLI, 15% of leaks were detected by contrast enema compared to 0.0% in the no DLI group.

### Subgroup analysis—locally advanced disease

A total of 456 (56.0%) patients met NCCN criteria for adjuvant therapy, including 24 diagnosed with a postoperative AL. Among these 456 patients, 254 (55.7%) received adjuvant therapy initiated at a median of 1.6 (IQR: 1.3–2.1) months postoperatively. Ten (42%) patients with AL received adjuvant therapy at a median of 1.9 (IQR: 1.5–2.1) months postoperatively, and 244 (56.5%) patients without AL received adjuvant therapy at a median of 1.6 (IQR: 1.3–2.0) months postoperatively. Controlling for the competing risk of death, AL correlated with a lower probability of receiving of adjuvant therapy on univariate analysis (SHR: 0.65, 95% CI: 0.434–0.98, *p* = 0.04).

**Fig. 5:**
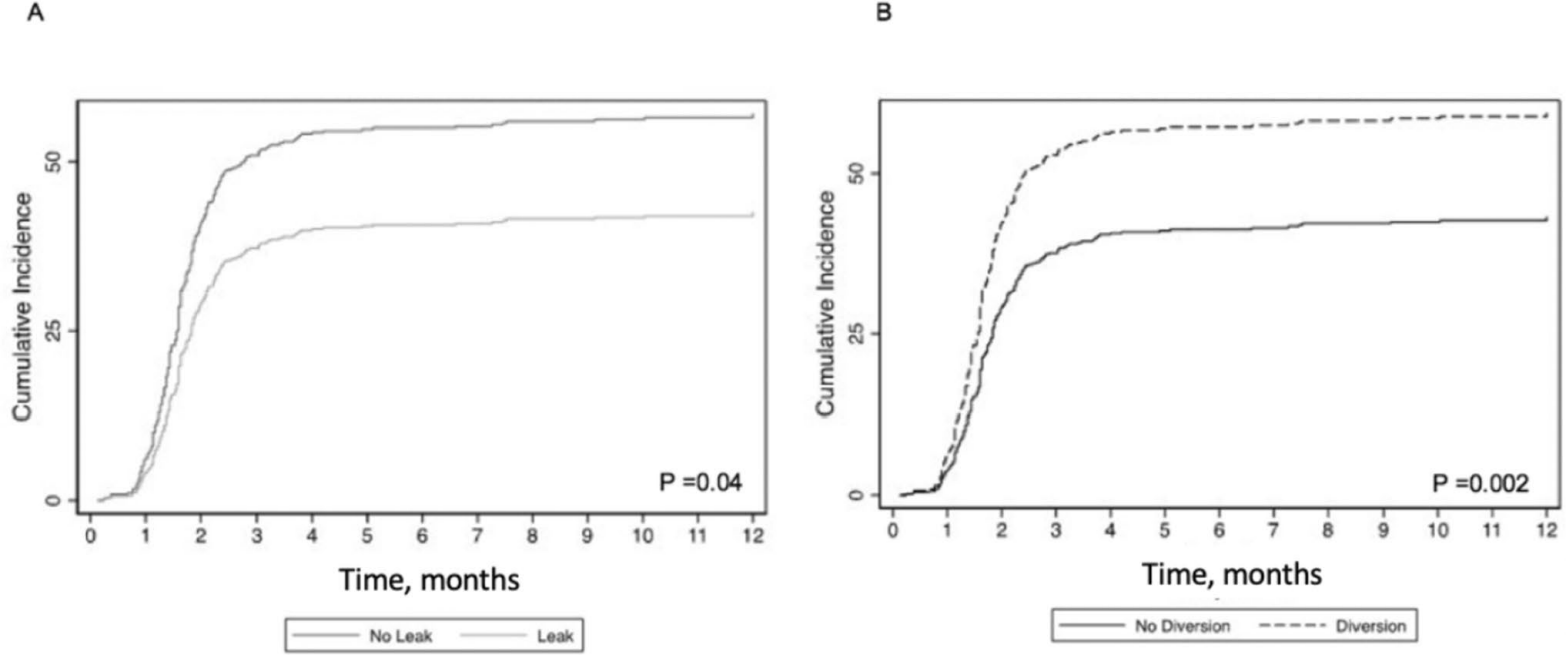
Competing risk regression for receipt of adjuvant therapy in patients with locally advanced rectal cancer. Receipt of adjuvant therapy, with mortality as a competing risk. **A** Receipt of adjuvant therapy by presence or absence of AL. The light gray line represents patients with AL, and the dark gray line represents patients without AL. **B** Receipt of adjuvant therapy by presence or absence of diverting loop ileostomy at index operation*.* The dashed line represents patients with diverting loop ileostomy at the time of resection, and the solid line represents patients without diverting loop ileostomy at the time of resection

Among patients meeting the criteria for adjuvant therapy, 332 (72.3%) patients had a LAR with initial DLI. Patients with LAR with initial DLI were more likely than those without a DLI to receive adjuvant therapy on univariate analysis (DLI: 59.6% vs. No DLI: 40.5%; *p* < 0.001). For those receiving adjuvant therapy, time to treatment, in months, was similar (DLI: 1.6 [IQR, 1.3–2.1] vs. No DLI: 1.4 [IQR: 1.2–2.1]; *p* = 0.14). Controlling for the competing risk of survival, DLI was significantly correlated with an increased receipt of adjuvant therapy for patients with and without AL on univariate analysis (SHR: 1.59 [95% CI: 1.19–2.14]; *p* = 0.002) (Fig. 5). On multivariable analysis, the trends between receipt of therapy and AL as well as DLI continued, but significance was not achieved (Table 3). There were no differences in 3-year OS (log rank: *p* = 0.83) (Fig. 3B) or recurrence with mortality as a competing risk (SHR: 1.08, [95% CI: 0.70–1.68]; *p* = 0.723) between patients with or without AL in this subgroup.

**Table 3:** Factors related to the receipt of adjuvant therapy controlling for the competing risk of death

	Multivariate analysis A			Multivariate analysis B		
	SHR	95% CI	*p*-value	SHR	95% CI	*p*-value
Anastomotic leak (ref = no leak)	0.7	0.45–1.09	0.12	0.68	0.46–1.01	0.06
DLI (ref = no DLI)	1.5	1.09–2.06	0.01	1.28	0.93–1.76	0.13
Age	0.97	0.97–0.98	** < 0.001**	0.97	0.97–0.98	** < 0.001**
Male sex (ref = female sex)	1.04	0.68–1.58	0.86	1.01	0.72–1.44	0.94
Weight loss (ref = no preoperative weight loss)				0.86	0.64–1.16	0.32
Neoadjuvant CRT (ref = no neoadjuvant CRT)				0.71	0.58–0.88	0.002
Active smoking (ref = non-smokers)				1.37	1.25–1.50	** < 0.001**

Bolded values indicated statistical significance

*DLI* diverting loop ileostomy, *SHR* subhazard ratio, *CI* confidence interval, *CRT* chemoradiation, *ref* reference

## Discussion

In this multi-institutional analysis of risk factors for anastomotic leak, we demonstrate that male sex is strongly associated with AL while fecal diversion is protective when controlling for other clinically important factors. Furthermore, we show fecal diversion significantly influences the natural history of patient’s suffering from AL, with a higher rate of operative intervention in non-diverted patients. The time to diagnosis of AL in DLI patients tended to be longer, likely in part, to a proportion of clinically asymptomatic leaks being found on contrast enemas performed prior to ostomy reversal. Interestingly, we demonstrate that the presence of fecal diversion at index operation is positively associated with receipt of adjuvant chemotherapy when controlling for the competing risk of death in appropriate patients. This trend persists when controlling for clinically relevant variables, although it loses statistical significance. This may be secondary to the small numbers of patients with AL in our cohort and warrants future investigation. Despite these differences in perioperative outcomes and receipt of adjuvant therapy, there was no difference in the long-term oncologic outcome of overall survival between cohorts.

The anastomotic leak in this study of 4.66% is lower than that reported in other large studies which range between 10 and 20% depending on the length of follow-up [3, 23]. However, it is worth noting that some recent database studies have reported similar leak rates in the range of 3–8% [24, 25]. One potential reason for this lower leak rate and limitation of our study is the inability to classify perianastomotic abscesses without signs of active leak as AL in our analysis. While we did classify pelvic abscess with a strong suspicion for AL as AL, several events where AL was less suspected may have not been included. These pelvic abscesses associated with the anastomosis are considered AL in the International Study Group of Rectal Cancer definitions, but this data was not reliably available [26]. Additionally, as contrast enemas were not routinely performed in patients without DLI, subclinical leaks in this patient population may have been unaccounted for. Despite these limitations, we feel that this real-world granular data is interesting and affords insight to clinically relevant AL outcomes.

Male sex is consistently associated with an increased risk of AL in rectal anastomoses, which has historically ascribed to a narrow pelvic inlet and resultant technical challenges during anastomosis creation [5, 27–30]. However, male leak rates have been higher in all colorectal anastomoses and have had higher leak rates when controlling for pelvic dimensions indicating that hormonal differences may place a significant role in AL [31–33]. More concerning, there is evidence to suggest that men are more likely to develop pelvic sepsis with AL, leading some authors to recommend increased consideration of routine protective stoma in male patients [29]. Similarly, we demonstrated an association of unintentional preoperative weight loss with AL. Malnutrition has been correlated with AL in multiple studies of LAR for rectal cancer with experts advocating for early identification and intervention for this modifiable risk factor [34–36].

Our analysis adds to the growing body of literature suggesting that fecal diversion at the time of index operation decreases the risk and adverse sequelae of AL [7, 8, 37, 38]. A 2007 study by Matthiessen et al. randomized 234 patients to defunctioning loop stoma or no stoma during rectal resection and found an increased rate of overall and symptomatic AL in the nondiverted group [7]. In addition to echoing these results, we identified additional contributing factors for AL. Identifying risk factors and instituting appropriate mitigation efforts are important given the reported association of AL with permanent stoma, long-term bowel dysfunction, and decreased quality of life [2, 39]. Furthermore, the granularity of our data provides a detailed illustration of the range of clinical presentations among patients with AL and demonstrates critical differences between patients with and without DLI during resection. Non-diverted patients were diagnosed and treated for leak earlier, overwhelmingly diagnosed by computed tomography, and more likely to undergo operative intervention. These characteristics are consistent with a more acute, severe presentation often accompanied by instability, peritonitis, or pelvic sepsis. Diverted patients with AL presented later and, in most cases, were successfully managed with non-operative treatment and drain placement. Leak diagnosis by contrast enema during evaluation for stoma reversal suggests a subclinical presentation in several of these patients. Prior studies have similarly identified fecal diversion as a key factor mediating the severity and timing of clinical presentations and outcomes following AL [30, 40]. A recent study by Rutegård et al. demonstrated very similar patterns in the timing of AL in patients with or without fecal diversion [41].

We demonstrated a trend towards increased receipt of adjuvant chemotherapy in patients with DLI and locally advanced rectal cancer. This finding may, in part, be explained by increased rates of reoperation and infectious complications in non-diverted patients; however, there is very little published data regarding risk factors for non-receipt of adjuvant therapy [42]. Taken together, these data demonstrate reduced frequency and severity of AL and increased likelihood of appropriate adjuvant therapy in patients with DLI which may further support universal fecal diversion during LAR for rectal cancer [43]. These differences, however, failed to translate into improved long-term oncologic outcomes such as recurrence or survival (*p* = 0.052). The reported effect of AL on oncologic outcomes in the literature is conflicting [4–6]. There is also no high-level evidence that demonstrates improved survival in rectal cancer with the receipt of adjuvant chemotherapy [44–46].

Additionally, as with all operative interventions, the creation of DLI is not without complications and drawbacks. The estimated morbidity associated with stoma creation from the time of creation to reversal, including complications following reversal, is approximately 15% and 18%, respectively, and includes dehydration and kidney injury [11–14, 47–49]. Furthermore, protective ostomies are permanent in some estimated 25–35% of cases and may increase rates of readmission and predispose to worse adjuvant therapy-associated complications [15, 50]. The authors of this study therefore support liberal use of DLI in patients where the predicted risk of AL exceeds the risk of stoma-associated morbidity. The difficulty in implementing this strategy lies in estimating these relative probabilities. This study therefore highlights an ongoing need for validated prediction models and clinical risk scores. These assessments should ideally reflect current practice and include high-quality risk assessment of patients receiving total neoadjuvant therapy. While the present neoadjuvant treatment transition to total neoadjuvant therapy would mitigate the effects of AL on receipt of adjuvant treatment, the prolonged hospital stays, readmission, and need for early operative intervention in AL patients would be diminished. Additionally, ultimate ostomy reversal may be improved with DLI at the index operation.

This study has several limitations. The retrospective nature of this analysis precludes any conclusions regarding causality or the direction of observed associations. Associations in models may be biased by data missingness and excluded variables. Variables with significant missing data in this study were removed from model consideration instead of using multiple imputations given that the missingness was not assumed to be random and could be associated with a patient’s clinical condition [51]. Variables not collected in the database, for example, abscess location discussed previously, confound the findings in this study. There is a degree of selection bias as data was primarily obtained from tertiary centers and may not reflect findings at community-based institutions. The exploratory nature of this analysis may predispose to an increased risk of false positive findings (subclinical AL); however, the granular nature of the database allows for substantive support of the results. Unfortunately, no data were collected regarding factors influencing the decision to create a diverting stoma at the time of LAR, limiting any efforts to determine if these factors were similar or dissimilar to those associated with the presence of AL. Additionally, no data were collected regarding the decision to maintain or reverse diverting stomas. The reasons for non-reversal of stoma in patients who underwent stoma creation during the initial operation or as treatment for AL were therefore not elucidated in this study. Due to the limited number of events, the time-varying component of AL as it relates to receipt of therapy was not accounted for, which may affect the accuracy of point estimates.

## Conclusion

This study identifies fecal diversion at rectal cancer resection as a modifiable protective factor against AL. The strong protective association of fecal diversion noted on multivariable analysis and its attenuation of adverse clinical outcomes in the context of AL should be further studied. This finding, combined with a trend in increased likelihood of adjuvant therapy in appropriate patients, suggests that protective stoma should be strongly considered at the time of rectal cancer resection, especially in males and patients with locally advanced disease at increased risk for AL.

## Data Availability

The raw data and statistical code that support the findings of this study are available from the corresponding author, JHM, upon reasonable request.
